# Case series: O-arm navigation assisted by the Wiltse approach improves the accuracy of pedicle screw placement in ankylosing spondylitis combined with thoracolumbar fractures

**DOI:** 10.1097/MD.0000000000036807

**Published:** 2023-12-29

**Authors:** Chang-Ming Li, Shi-Jie Zhao, Jian-Zhu Xu, Qiang Li, Ren-Fu Quan, Xiao-Mei Deng

**Affiliations:** a Department of Orthopaedics Institute, Xiaoshan Traditional Chinese Medical Hospital, XiaoShan District, Hangzhou, Zhejiang Province, China.

**Keywords:** ankylosing, fracture fixation, internal, navigation, pedicle screws, spinal fractures, spondylitis

## Abstract

Here we assessed the accuracy of O-arm navigation assisted by Wiltse approach to improve based pedicle screw insertion in ankylosing spondylitis combined with thoracolumbar fractures. We then compared it with the freehand pedicle screw insertion technique. The study sample included 32 patients with ankylosing spondylitis combined with thoracolumbar fractures. Pedicle screw reduction and internal fixation was performed under an O-arm navigation system assisted by a Wiltse approach-combined osteotomy (“navigation group,” n = 17) and posterior pedicle screw reduction and internal fixation was performed using freehand technique combined osteotomy (“freehand group,” n = 15). We then compared the operation time and bleeding volume between the 2 groups. The visual analog scale (VAS) and Oswestry disability index (ODI) were then used to evaluate the clinical efficacy and the kyphosis Cobb angle was used to evaluate the radiological efficacy before operation, 3 days after operation and after the last follow-up. All complications were noted when detected. Finally, classification of screw positions as proposed by Neo et al was used to evaluate the relationship of the position between the screw, the bone cortex, and the incidence of screw penetration. All patients were followed up for 18 to 36 months (i.e., 24.2 ± 3.5 months). The operation time and intraoperative bleeding volume of the navigation group were significantly shorter (lower) than those of the freehand group (*P* < .05). In addition, Both groups showed significantly decreased VAS, ODI, and Cobb angle 3 days after the operation and at the last follow-up when compared to values recorded pre-operation. However, we found no significant difference in VAS, ODI, and Cobb angle between the 2 groups (*P* > .05). We identified no complications (e.g., infection, VTE/PE, or nerve injury). Moreover, the pedicle screw placement position of the navigation group was better than that of the freehand group (*P* < .05), and the screw cortical penetration rate was lower than the freehand group (*P* < .05). During the process of posterior pedicle screw placement, O-arm navigation assisted by the Wiltse approach can significantly reduce operation time, minimize the amount of bleeding volume, and enhance the accuracy of pedicle screw implantation.

## 1. Introduction

Ankylosing spondylitis (AS) results in gradually increased kyphosis over time due to bone fusion and kylosis deformity.^[1]^ Moreover, once spinal segment motion is reduced, a slight external force can cause thoracolumbar fracture.^[2]^ Because AS combined with thoracolumbar fracture is similar to long bone fractures, the fractured end is unstable and often requires surgical treatment.^[3,4]^ Strong internal fixation is the gold standard for treating long bone fractures. Moreover, due to their biological performance and high strength, pedicle screws can provide strong internal fixation for AS combined with thoracolumbar fracture. However, AS shows “bamboo-like” changes after spinal segment fusion; thus, when the anatomical landmarks are not clear there is a risk of nerve damage occurring during freehand nailing. In addition, the vertebral pedicle of the upper thoracic vertebra is small, and fluoroscopy is easily affected by the ribs and scapula. This risks penetration of the cortex into the spinal canal when the pedicle screw is inserted, which can cause serious complications. Therefore, improving the accuracy of nail placement has become an important problem for spine surgeons. With the development of modern technology and the concept of minimally invasive surgery, the O-arm is used more and more often during orthopedic surgery, since it facilitates intraoperative three-dimensional CT imaging and intraoperative real-time navigation, both of which greatly improve the accuracy of the screwing procedure.^[5–7]^ However, to date there are few reports on O-arm navigation-assisted pedicle screw placement for AS combined with thoracolumbar vertebral fractures. The purpose of this study was therefore to examine the efficacy of an O-arm navigation-assisted Wiltse approach relative to a freehand posterior pedicle screw combined osteotomy in cases of AS combined with thoracolumbar fracture.

## 2. Clinical data and methods

### 2.1. Population

After approval from the institutional review board, a retrospective case–control study was conducted in patients with AS combined with thoracolumbar fracture who were surgically treated at our spinal medicine center between January 2013 and December 2017.

The following inclusion criteria were used for patient inclusion in the study: (1) a long-term medical history of AS^[8,9]^; (2) combined thoracolumbar fracture (single segment); (3) a Cobb angle of ≥20° for kyphosis deformity; (4) complete clinical data; (5) preoperative evaluation with no contraindications for surgery, and consent to plan treatments. Exclusion criteria were as follows: (1) patients with a previous history of thoracolumbar surgery; (2) incomplete imaging data; (3) patients who could not tolerate surgery by preoperative evaluation; and (4) cases involving old fractures.

A total of 32 patients were divided into 2 groups. The first group (“navigation group”) comprised 17 patients (2 female and 15 male; aged 38–67 years, mean age: 49.7 years) who received O-arm navigation system assisted by a Wiltse approach-combined osteotomy. The cause of injury for these patients was as follows: 9 cases of traffic injury, 6 cases of injury by falling from a height, and 2 cases of ordinary fall injury. Injury segments were as follows: 1 case of T1–4 injury, 7 cases of T5–10 injury, and 9 cases of T11–L2 injury. These cases also included 2 cases of spinal cord injury with an American Spinal Injury Association classification of grade D. The second group (“freehand group”) comprised 15 patients (1 female and 14 male; aged 40–71 years, mean age: 50.4 years) who received traditional freehand posterior pedicle screw reduction internal fixation combined osteotomy. Here, the causes of injury were as follows: 8 cases of traffic injury, 4 cases of injury by falling from a height, and 3 cases of ordinary fall injury. Injury segments were as follows: 2 cases of T1–4 injury, 1 case of T5–10 injury, and 12 cases of T11–L2 injury. This group included 3 cases of spinal cord injury with an American Spinal Injury Association classification of grade D). Table 1 lists their main features.

**Table 1:** Patients’ clinical and radiological features*.

Parameter	Navigation group	Free-hand group
Demographic data		
No. of patients	17	15
Sex of patients	2F/15M	1F/14M
Age at diagnosis	49.7 years (range: 38–67)	50.4 years (range: 40–71)
Cause of injury		
Traffic injury	9 (52.95%)	8 (53.33%)
High fall injury	6 (35.29%)	4 (26.67%)
Fall injury	2 (11.76%)	3 (20%)
Injure segment		
T1–4	1 (5.88%)	2 (13.33%)
T5–10	7 (41.18%)	1 (6.67%)
T11-L2	9 (52.94%)	12 (80%)
Spinal cord injury		
Yes	2 (11.76%)	3 (20%)
No	15 (88.24%)	12 (80%)
Time from injury to surgery [days]	3.1 (range: 0–5)	3.0 (range: 0–5)

* Data are shown as mean ± SD or n (%) unless otherwise indicated.

### 2.2. Surgical procedure

Trained operators performed all procedures in an operating room under general anesthesia. This procedure used a pedicle screw internal fixation system (Beijing Fulle Company). An O-arm navigation assistance system (Medtronic Company) was used during treatment of the navigation group.

### 2.3. Navigation group

Patients first received general anesthesia with air duct intubation while lying in the prone position with a silicone pillow on both sides. The abdomen was then suspended, and spinal cord function was monitored by somatosensory evoked potentials and motor evoked potentials. After perspective positioning, the median incision was made along the center to cut through skin and subcutaneous tissue until the chest (waist) or back fascia were visible. We then made about 2 cm longitudinal cut, along the spine in the fascia, along the multifidus from the longest muscle gap. This revealed bilateral articular process and the navigation reference frame was then fixed in the spine. This involved O-arm navigation for the three-dimensional imaging system, import system, and intraoperative registration, and used a navigation opening and probe to guide the screw with respect to direction and depth. After opening, the screw path was gradually expanded by expanding the vertebral body, and the screw path was inserted with a navigation probe to examine the bottom of the screw path and any damage around it. After screw placement, we scanned the inserted screw area again using the O-arm system to confirm that the position of the screw was correct. Intraoperative osteotomy angle (i.e., PSO osteotomy) was confirmed according to the preoperative kyphosis Cobb angle and the connecting rod was then fixed. Next, the wound was flushed with saline, the drain was placed, and the wound closed layer by layer.

### 2.4. Freehand group

For the freehand group, we used the same position and anesthesia method as above. However, after the median approach, we revealed the tissue under the periosteal stripping bilateral sacral spinous muscle and pulled it to both sides to reveal the corresponding segment of the spike and the vertebra. Next, we identified the transverse midpoint horizontal line and injury joint outer edge intersection to plan the injection point, using a double pedicle drill and a positioning needle. We then used X-ray fluoroscopy positioning, an expanding spike, and a clear probe spike. Once we confirmed there was no damage, the screw was inserted using parallel X-ray clear screw placement. Once again, the intraoperative osteotomy angle (PSO osteotomy) was confirmed according using the preoperative kyphosis Cobb angle and the connecting rod was fixed. The wound was closed as described above.

### 2.5. Postoperative management

Prophylactic antibiotics were administered for 24 to 48 hours with the patient in the supine or lateral position. Afterward, the patient was instructed to turn over and beat their back lightly to strengthen the muscle contraction function of both lower limbs, and to prevent venous thrombosis by removing <50 mL samples of drainage fluid. Patients were able to leave bed approximately 1 week after surgery. X-ray images and CT scans were then reviewed at 3 days, 1 month, 6 months, and 12 months post-surgery as well as at a final follow-up.

### 2.6. Clinical assessment

Next, the time of surgery and the degree of intraoperative bleeding volume were compared between the 2 groups. Specifically we assessed the visual analog score (VAS), neurological dysfunction index (Oswestry disability index [ODI]) score, and Cobb angle at preoperation as well as 3 days post-operation and at the last follow-up. All postoperative complications were then observed. After postoperative CT examination, the screw position grade, and cortical penetration were evaluated using the Neo grading evaluation system. Specifically, Neo Grade 4: Grade 0, the pedicle screw did not puncture the pedicle cortex; Grade 1, the pedicle screw punctured the cortex, but this puncture wound was less than < 2 mm in depth; Grade 2: the pedicle screw puncture cortex was >2 mm but <4 mm in depth; Grade 3: the pedicle screw puncture cortex was >4 mm.

### 2.7. Statistical analysis

Data are expressed as mean ± standard deviation and all statistical analyses were performed using SPSS version 26.0 (IBM SPSS, Armonk, NY). Paired Student *t* tests and Dunnett *t*-tests were used to evaluate changes in structured data at different times. In addition, the VAS score, Cobb angle in the sagittal plane, and count data were all expressed as percentages, and comparisons between groups were performed using a χ^2^ tests. For all statistical comparisons, a *P*-value of <.05 was considered to be statistically significant.

## 3. Results

**Figure 1.**
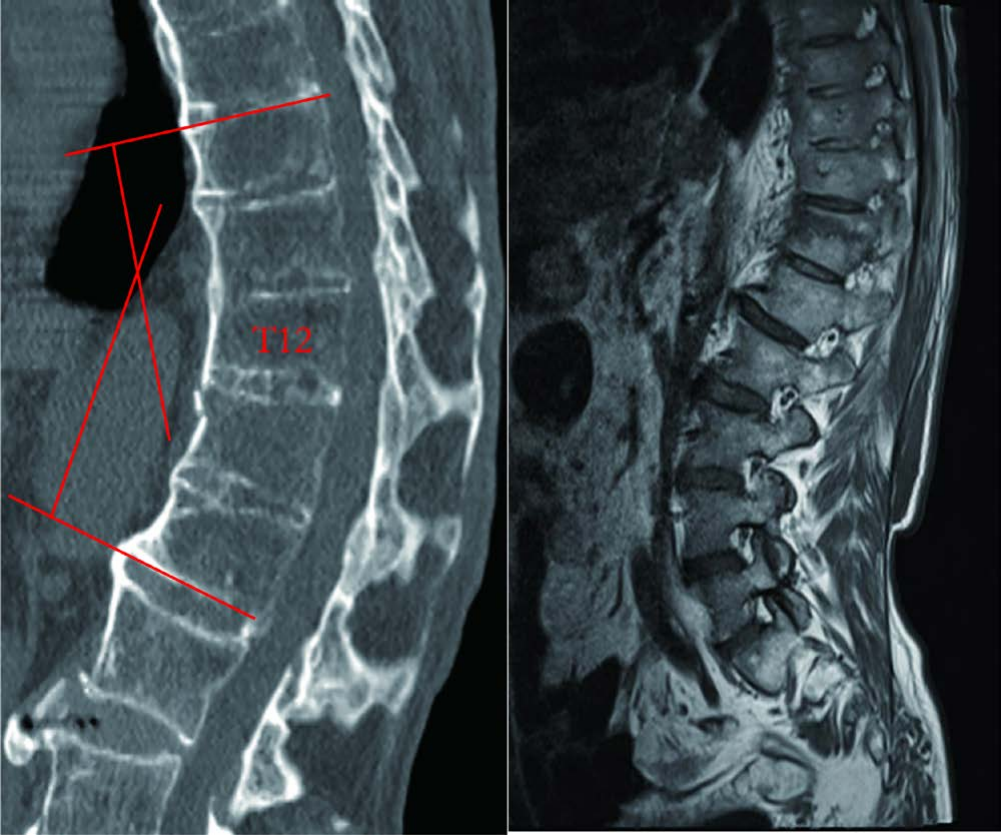
A 61-year-old patient AS with T12 vertebral compression fracture was treated with O-arm navigation assisted by wiltse approach internal fixation combined osteotomy. The pre-operative kyphosis Cobb angle is 38.6 degrees. CT indicated “bamboo” changes in the spine (on the left). MRI indicated T12 vertebral compression fracture (on the right left).

**Figure 2.**
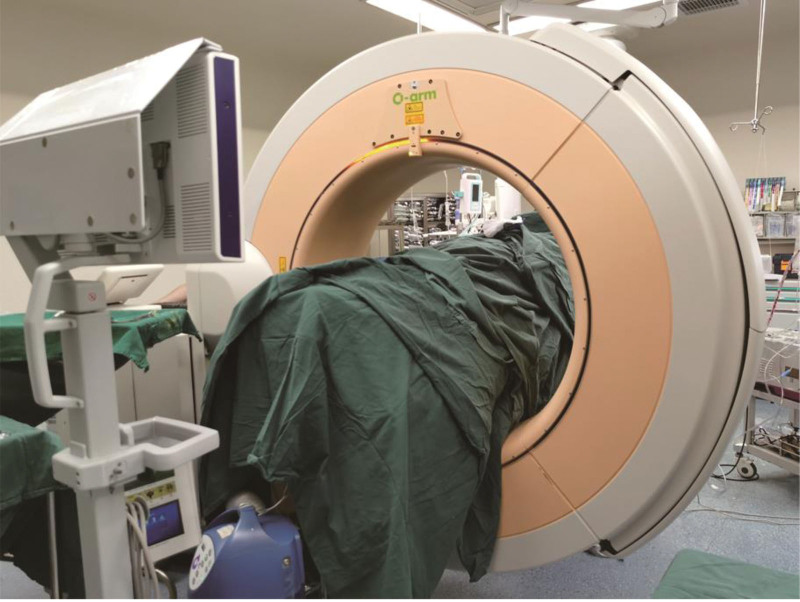
Intraoperative O-arm navigation.

**Figure 3.**
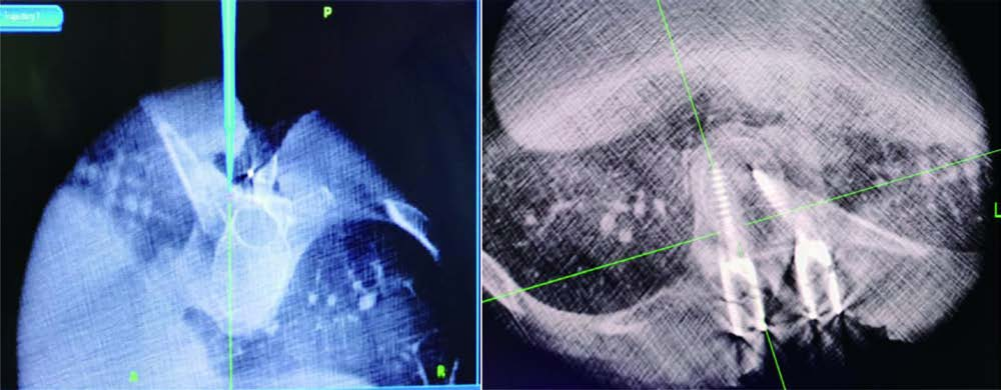
Determine needle placement and screw placemen under O-arm navigation.

**Figure 4.**
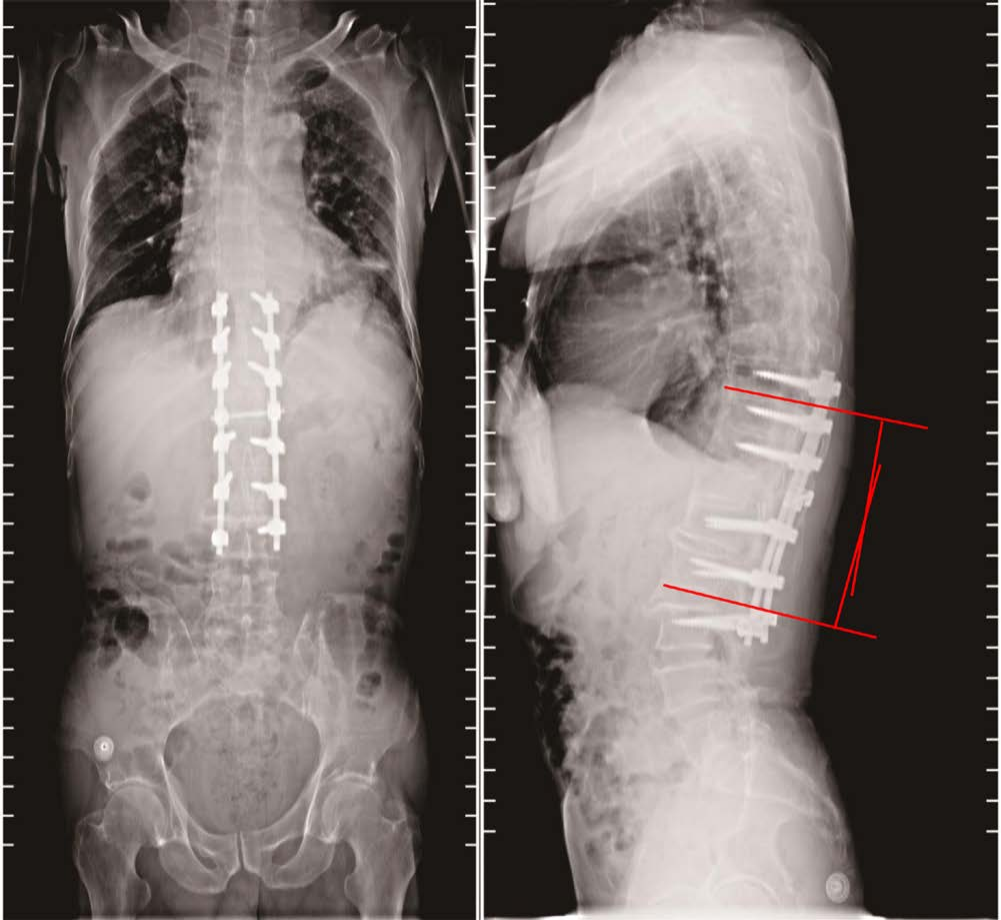
X-ray images of spine after surgery. The post-operative kyphosis Cobb angle is 7.7 degrees.

**Figure 5.**
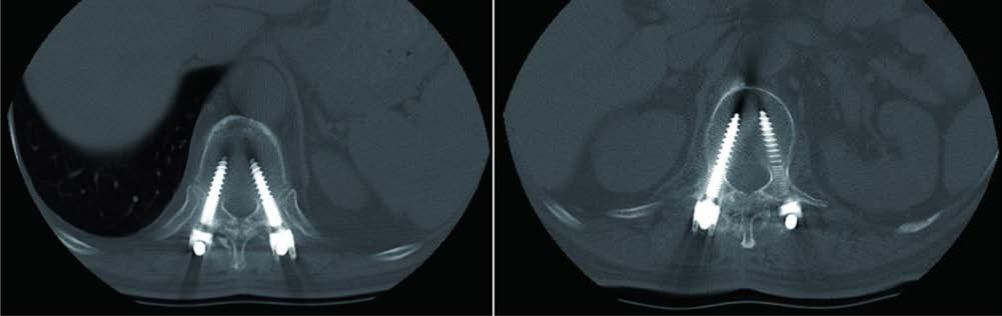
Postoperative X-ray scans show that screw position and titanium rod length are suitable (Grade 0) (on the left side is T11, on the right side is L1).

All patients were followed up for 18 to 36 months (mean: 24.2 ± 3.5 months). The mean operation time of the navigation group was 148.6 ± 8.5 minutes, which was significantly shorter than the 189.7 ± 8.4 minutes mean time of the freehand group (*P *< .05) (Table 2). Moreover, the mean bleeding volume of the navigation group was 569.4 ± 83.9 mL, which was significantly less than the mean of 766.7 ± 108.7 mL of the freehand group (*P *< .05) (Table 2). Both groups showed significantly decreased VAS and ODI 3 days after the operation and at the last follow-up when compared to values recorded pre-operation. However, we found no significant difference in VAS or ODI between the 2 groups (*P* > .05). In addition, both groups showed significant decreases in kyphosis Cobb angle 3 days after operation and at last follow-up relative to values recorded pre-operation. However, we found no significant difference in kyphosis Cobb angle between the 2 groups (*P* > .05) (Table 3). No severe complications such as infection, VTE/PE, or neurovascular injury were recorded for patients in either group. A total of 208 screws were placed in the navigation group (i.e., Neo Grade 0: 205 screws, Grade 1: 3 screws), while 182 screws were placed in the freehand group (i.e., Neo Grade 0: 168 screws, Grade 1: 10 screws, Grade 2: 3 screws, Grade 3: 1 screw; *P *< .05). The incidence of cortical penetration was 1.4% (i.e., 3/208) in the navigation group and 7.7% (14/182) in the freehand group (*P *< .05) (Table 4). Figures 1–5 shows a representative case; this represents a 61-year-old male patient with AS combined with thoracolumbar fractures who underwent O-arm navigation assisted by Wiltse approach pedicle screw reduction.

**Table 2:** Comparison of surgical time and intraoperative bleeding (measured as mean ± SD).

Parameter	Navigation group	Free-hand group	Statistical significance
Number of patients	17	15	
Surgical time (min)	148.6 ± 8.5	189.7 ± 8.4	*P* = .0000000015
Intraoperative bleeding (mL)	569.4 ± 83.9	766.7 ± 108.7	*P* = .000047

**Table 3:** Variations of each measured parameter preoperatively, postoperatively, and at latest follow-up (measured as mean ± SD).

Parameter	Navigation group	Free-hand group	Statistical significance
Number of patients	17	15	
VAS			
Preop	7.2 ± 0.8	7.2 ± 0.7	*P* = .891
Postop 3d	2.8 ± 0.7	2.7 ± 0.7	*P* = .897
Final follow-up	1.8 ± 0.7	1.9 ± 0.8	*P* = .690
ODI			
Preop	76.1 ± 3.3	75.8 ± 2.4	*P* = .309
Postop 3d	22.2 ± 2.7	21.9 ± 2.5	*P* = .335
Final follow-up	23.9 ± 2.4	24.5 ± 2.3	*P* = .787
COBB, angle			
Preop	37.2 ± 4.5	36.8 ± 4.0	*P* = .788
Postop 3d	7.9 ± 2.6	8.1 ± 2.9	*P* = .851
Final follow-up	9.8 ± 2.2	10.1 ± 2.3	*P* = .666

**Table 4:** Comparison of neo grading evaluation (measured as mean ± SD).

Parameter	Number of patients	Screw position sizing (piece)				Cortical penetration rate of (%)	
		Grade0	Grade 1	Grade2	Grade 3	Yes	No
Navigation group	17	205	3	0	0	1.4 (3/208)	98.6 (205/208)
Free-hand group	15	168	10	3	1	7.7 (14/182)	92.3 (168/182)
Statistical significance		*P* = .021				*P* = .003	

## 4. Discussion

AS with thoracolumbar fracture involves 3 columns^[10]^ and is an unstable fracture that is suitable for early surgical treatment.^[11–13]^ Pedicle screw internal fixation is a classic fixation approach to posterior thoracolumbar injury, and shows good biological performance and high holding strength. However, patients with AS paravertebral ligament calcification ossification have been found to show “bamboo-like” changes in which the joint space disappears, bone ankylosis emerges, the joint becomes fuzzy, and kyphosis deformities occur., This is thought to be due to the screw not finding the “herringbone ridge,” which results in multiple screw attempts that make it easy to cause rupture of the opening cortex. Thus, there is a need to change the needle position and increase the pedicle screw tilt angle, which can cause the pedicle screw to reach the spinal canal and cause nerve damage. With the continuous innovation of surgical technology, O-arm navigation assistance has become increasingly common in spinal surgery. This study shows that the O-arm navigation aid technology can effectively improve the accuracy of screw placement.^[5]^ Given the rapid rehabilitation and minimally invasive surgery concepts, the Wiltse approach can not only reduce tissue damage, but also play a positive role in reducing blood loss and increasing quality of life.^[14,15]^ Here, a retrospective case-control study of 32 patients with AS combined with thoraco-lumbar fracture showed that pedicle screw reduction with O-arm navigation assisted by the Wiltse approach could significantly shorten operation time, reduce the amount of intraoperative bleeding volume, and improve the accuracy of pedicle screw placement.

The treatment principles for AS combined with thoracolumbar fracture mainly include satisfactory reduction, strong fixation, and reconstruction stability. Using posterior pedicle screw internal fixation can effectively maintain spinal stability and provide a good foundation for fracture healing. In this study, 17 patients with AS and thoracolumbar fracture used a Wiltse-assisted internal fixation with pedicle screw assisted by O-arm navigation, and this was found to significantly improve pain symptoms and achieved a good orthopedic effect. Compared to traditional freehand nail placement, the operation time was shortened, the amount of bleeding was reduced, and screw placement was significantly more accurate.

The treatment of AS combined with thoracolumbar fracture by the Wiltse approach assisted by O-arm navigation has several advantages, listed below: (1) Improved screw placement and surgical safety. Traditional freehand nailing needs to be completed under X-ray monitoring, and the blurred needle insertion point in AS patients, requires multiple fluoroscopies, and has high pin error rate and a steep learning curve. O-arm navigation assistance technology can provide two- and three-dimensional imaging, and track surgical instruments in real time to guide the placement of pedicle screws; (2) Reduced radiation exposure to medical personnel. The O-arm procedure needs less radiation-based imaging and a shorter surgery, which reduces the intraoperative radiation exposure time for patients and surgeons. It may also, to a certain extent, reduce iatrogenic damage. In addition, Zwingmann et al,^[16]^ found that a three-dimensional navigation radiation dose of 822 ± 164 cGy/cm² was significantly lower than the conventional dose of 1843 ± 1 052 cGy/cm². (3) Mispositioned screws can be corrected in real time. Ruoyu et al^[17]^ found that 1 Neo Grade 3 screw was misplaced when using O-arch navigation, and a revision surgery was performed immediately (also with O-arm navigation), and the patient was Neo Grade 0 when imaged using a postoperative CT. In this study, 3 Neo Grade 1 cases were found in the navigation group without adjustment, and at postoperative CT only 1 Neo Grade 3, which was not revised since no neurological symptoms appeared at follow-up. Moreover, since O-arm navigation assistance can help surgeons adjust the screw position in real time, the penetration rate of the cortex in the navigation group (i.e., 1.4% or 3 of 208) was significantly lower than in the freehand group (i.e., 7.7% of 11 of 182). (4) Shortened operation times and reduced bleeding. After traditional wound opening, the median approach requires stripping away all layers of tissue, including bilateral sacral spinous muscle and others. Electrocoagulation is often needed to completely stop the bleeding and permit the surgeon to see well, and this extends the operation time. The Wiltse approach is to blunt separation between the multifidus muscle and the longest muscle space, which results in less tissue damage and less dissection. In the actual operation, to find the precise injection point, the freehand-place screw often attaches to vertebral solidified tissue and osteophytes, which also extends the operation time and increases the amount of bleeding.

There is no unified standard for the treatment of AS with thoracolumbar fracture assisted by O-arm navigation. However, this procedure mainly includes: (1) two- or three-column fractures of the intervertebral space or vertebral body, with or without kyphotic deformity; (2) vertebral rotation and vertebral deformity; (3) manual reduction of fracture dislocation; (4) fusion of the upper and lower joints if the screw point cannot be identified; (5) X-ray fluoroscopy-based visualization of the pedicle shadow. Some studies have shown that O-arm navigation can be safer and more accurate during the placement of pedicle screws with a smaller diameter.^[18]^ If the indications are properly selected, a satisfactory curative effect can be obtained. AS combined with thoracolumbar fractures is complex, and may involve more anterior and middle two- or three-column fractures, fracture segment instability, increased local stress of short segment fixation, and increased risk of long-term screw loosening and adjacent vertebral disease.^[19]^ Therefore, previous studies have suggested that posterior fixation can be extended up or down by 1 or 2 segments.^[20]^ Studies by Kleck^[21]^ and Weiguo et al^[22]^ found that the accuracy of O-arm navigation aids reached 96.8% and 94.2%, respectively, which avoided the need for multiple screws to reduce the risk of screw loosening. In this study, 17 cases of AS with thoracolumbar fractures were included and 208 screws were inserted, which yields an average of 6 segments placed per patient. Moreover, the screw cortical penetration rate was 1.4%. Finally, AS with thoracolumbar fractures is often accompanied by kyphotic deformity, but effective osteotomy can correct the spinal sagittal sequence. Here, the injured vertebra was generally not inserted, which also achieved a good effect.

During the operation, the following points should be noted: (1) the operating table should be adjusted before the operation to ensure that the O-arm navigation system can transmit the entire screw placement area; (2) The reference frame should be placed at the nearest or most distal end of the screw placement area to avoid intraoperative failure of the navigation probe due to reference frame occlusion; (3) the reference frame should be firmly fixed, The intraoperative operation should be gentle, and image drift-which is caused by intraoperative accidental collision-should be avoided; (4) if the fixed segment is longer, two or more three-dimensional CT images should be taken. When placing the screw, surgeons should consult the image of the corresponding part, and multiple passages of the machine should be avoided to decrease the risk of contamination of the surgical area and shorten the length of the procedure; (5) after placing the screw, the position of the screw should be confirmed using imaging. If necessary, the navigation system can be relocated to readjust the screw position.^[23]^ In our study, 17 patients required readjustment of the screw position after initial screw placement. Of these, 3 screws pierced the cortex, but all of these were shallow penetrations (i.e., <2 mm). Moreover, somatosensory evoked potentials and motor evoked potentials were monitored and registered as normal. In these cases the screw was not adjusted, and we observed no neurological complications at postoperative follow-up.

We also note that this study has several limitations. For instance, the number of patients included is relatively small, and the follow-up time was short. In future studies, further expanding the sample size and extending the follow-up time may provide valuable additional data. Moreover, O-arm navigation assistance technology is complex, requires professional technical personnel to operate, and has a steep learning curve. Moreover, slight collisions between the reference frame and the operator can cause slight differences in screw placement, making human error an important risk that can reduce the accuracy of screw placement.

In conclusion, satisfactory clinical efficacy and recovery of spinal sagittal sequence for AS combined with thoracolumbar fracture, can be obtained by using O-arm navigation assistance through the Wiltse approach and freehand screw placement. However, the application of O-arm navigation assistance using the Wiltse approach can significantly shorten operation time, reduce the amount of bleeding, and improve the accuracy of pedicle screw placement.

## Acknowledgments

The authors would like to thank all participating patients, as well as the study nurses, co-investigators, and colleagues who made this trial possible.

## Author contributions

**Conceptualization:** Chang-Ming Li, Xiao-Mei Deng.

**Data curation:** Chang-Ming Li.

**Formal analysis:** Shi-Jie Zhao.

**Funding acquisition:** Shi-Jie Zhao.

**Investigation:** Shi-Jie Zhao.

**Methodology:** Jian-Zhu Xu.

**Project administration:** Jian-Zhu Xu, Qiang Li.

**Resources:** Qiang Li.

**Software:** Ren-Fu Quan.

**Supervision:** Ren-Fu Quan.

**Validation:** Ren-Fu Quan.

**Writing – original draft:** Xiao-Mei Deng.

**Writing – review & editing:** Xiao-Mei Deng.
